# Genotoxicity Studies of Titanium Dioxide Nanoparticles (TiO_2_NPs) in the Brain of Mice

**DOI:** 10.1155/2016/6710840

**Published:** 2016-02-29

**Authors:** Hanan R. H. Mohamed, Nahed A. Hussien

**Affiliations:** Zoology Department, Faculty of Science, Cairo University, Giza 12613, Egypt

## Abstract

Titanium dioxide nanoparticles (TiO_2_NPs) are excessively used and represent one of the top five most commonly used nanoparticles worldwide. Recently, various studies referred to their toxic potential on various organs using different treatment route. Male Swiss Webster mice were orally administrated TiO_2_NPs (500 mg/kg b.w.) daily for five consecutive days and then animals were sacrificed at 24 h, 7 days, or 14 days after the last treatment. The present results report that exposure to TiO_2_NPs produces mild to moderate changes in the cytoarchitecture of brain tissue in a time dependent manner. Moreover, Comet assay revealed the apoptotic DNA fragmentation, while PCR-SSCP pattern and direct sequencing showed point mutation of Presenilin 1 gene at exon 5, gene linked to inherited forms of the Alzheimer's disease. Therefore, from these findings, the present study concluded that TiO_2_NPs is genotoxic and mutagenic to brain tissue which in turn might lead to Alzheimer's disease incidence.

## 1. Introduction

Nanotechnology means a new set of technologies that are used to develop nanoscale structures and devices with one dimension size 1–100 nm with special properties utilized in commercial applications [1]. Nanotechnology promises a great contribution to humanity, but without appropriate assessment of risks and safely, public confidence in this expanding field will diminish [2].

Concern has been raised about the effect of nanoparticles exposure on human health [3, 4]. Titanium dioxide (TiO_2_) is a widely used industrial nanomaterial that was used in various products including sunscreens, lacquers, and paints [5]. Therefore, Nano-TiO_2_ (TiO_2_NPs) risk assessment should be an integral part nowadays in our modern society. Human exposure to TiO_2_NPs may occur during both manufacturing and use. The major routes of TiO_2_NP exposure that have toxicological relevance in the workplace are inhalation and dermal exposure. Oral exposure, as a nonmajor route, may occur from toothpaste, food colorants, and nutritional supplements that contain TiO_2_NPs. In a recent study by Weir et al. [6], they found that candies, sweets, and chewing gums contained the highest amount of TiO_2_ in the scale of <100 nm. In nanomedicine, intravenous or subcutaneous injection of TiO_2_ nanoparticulate carriers is a unique way to deliver TiO_2_NPs into the human body [7].

Evidence of TiO_2_NPs exposure genotoxicity has been previously researched within various studies, including micronuclei development, DNA damage, and* in vitro* mammalian chromosomal aberrations. Moreover, genotoxicity investigations of different nanomaterials were published in an openly available scientific literature from all biological models [8].

Because of their nanosize and unique properties, nanoparticles can enter the body and freely cross different biological barriers. Various studies have assessed that inhaled/injected nanoparticles enter systemic circulation and migrate to different organs and tissues [9, 10]; in turn they could accumulate and damage them, especially those sensitive to oxidative stress (OS).

Oberdorster et al. [11] and Sager et al. [12] reported that TiO_2_NPs (21 nm) caused a higher pulmonary inflammatory response than TiO_2_, because TiO_2_NPs enter the alveolar interstitium with a much greater amount than TiO_2_. Another study revealed that a small fraction of pulmonary TiO_2_NPs were able to access the blood circulation and reach extrapulmonary tissues such as liver and kidneys, at 28 days after instillation [13].

The brain is another organ that could be severely affected by nanoparticle induced oxidative stress (OS) due to its high energy demands, low levels of endogenous scavengers, and high cellular concentration of OS targets. Recent experimental studies indicate that nanoparticles can cross the blood-brain barrier [14] and enter the central nervous system of exposed animals in low numbers [10, 15].

Presenilin 1 (PSEN1) gene is one of four important genes that are linked to inherited forms of the Alzheimer's disease (AD). Previous studies reported that mutation of PSEN1 gene leads to alternation of the intramembranous cleavage of the *β*-amyloid precursor protein by *γ*-secretase enzyme. In turn, this altered *β*-amyloid precursor protein increased the production of A*β*_42_ that is accumulated chronically in some brain regions with very little production to fibril-rich amyloid plaques and few related neuritic and glial cytopathology; this leads to dementia. Selkoe [16] report in detail this hypothetical sequence of the pathogenetic steps of familial forms of AD.

The present study focuses on the histological, genotoxic, and mutagenic effect of TiO_2_NPs on mice brain cells using histological assay, COMET assay, detection of point mutation of PSEN1 gene (AD related gene) using SSCP evaluation followed by direct sequencing for mutated samples. Additionally, the present study aimed to know if there is a relation between TiO_2_NPs exposure and AD incidence.

## 2. Material and Methods

### 2.1. Animals

This study was performed on 12 mature male Swiss Webster mice, weighing about 25–30 g b.w. and aged 10–12 weeks. Animals were purchased from National Research Center animal house (Dokki, Giza, Egypt). Mice were housed for 7 days to be accommodated with our laboratory conditions. Food and water were presented* ad libitum*. Animals received care according to the criteria outlined in the “Guide for the Care and Use of Laboratory Animals.”

### 2.2. TiO_2_NPs and Their Characterization

The TiO_2_NPs used in this study were a mixture of rutile and anatase forms purchased from Sigma Chemical Co., (St. Louis, MO, USA) in the form of odorless and white powder in the nanoscale range <100 nm using Brunauer-Emmett-Teller (BET) method and <50 nm using X-ray diffraction method with a purity of 99.5% and CAS number 13463-67-7. As mentioned in our previous study [17], TiO_2_NPs were ultrasonicated in deionized distilled water using the biologics ultrasonic homogenizer (Model 150VT) immediately prior to characterization and administration and the pH value of TiO_2_NPs suspensions was 6.8 and characterized using X-ray diffraction (XRD) to identify the crystal phase and the average crystallite size. Indeed, the particle size and morphology of TiO_2_NPs suspensions were detected using transmission electron microscopy (TEM) and the dispersion and aggregation status of these nanoparticles in water were determined by the dynamic light scattering (DLS) method using particle size distribution and zeta potential analyzer (Zeta sizer Nano ZS90, Malven Instruments, UK).

### 2.3. Experimental Protocols

Mice were divided into four groups, 3 mice/group: Group 1: negative control group (untreated group); Groups 2, 3, and 4: animals daily were orally administrated TiO_2_NPs by oral gavage (500 mg/kg [18]) for 5 days and were sacrificed at 24 h, 7 days, and 14 days, respectively. After dissection, the brain tissues were excised for further evaluation.

### 2.4. Histopathological Evaluation

The brain was removed from the skull, and brain tissue portion was fixed in 10% neutral buffer formalin, washed with tap water, and dehydrated in a series of alcohols. The dehydrated tissue was cleared by using xylol and then embedded in paraffin wax at 60°C; blocks were cut at 5 microns using a microtome. Brain sections were stained using haematoxylin and eosin [19] for the investigation of general histological changes.

### 2.5. Molecular Evaluation

#### 2.5.1. DNA Extraction and PCR Amplification

Genomic DNA was extracted from brain tissue portion using the Genomic DNA Purification kit (Fermentas) according to the manufacturer's instructions. The quantity of DNA was estimated by absorbance reading at 260 nm and DNA shows more purity was estimated by ratio of absorbance reading between 260 and 280 nm.

For polymerase chain reaction (PCR), P1 forward 5′-aatctacaccccattcacag-3′ and reverse 5′-gcccccaactctcccacc-3′ were used to amplify exon 5 of PSEN1 gene (231 bp) of mouse [20]. The PCR reaction mixture was set up using sterile water, 100 ng/*μ*L of extracted DNA, 1 *μ*L forward/reverse primers (20 pmol/*μ*L), and 10 *μ*L 2x ready to use master mix (Fermentas) in a 0.2 mL PCR eppendorf tube. Cycling was started in the Thermal Cycler (Programmable Thermal Cycler, PTC-100TM thermal cycler, Model 96; MJ Research, Inc., Watertown, MA, USA), with initial denaturation at 94°C for 3 mins, denaturation at 94°C for 30 s, primer annealing at 60°C for 1 min, and then primer extension at 72°C for 1 min, for 35 cycles. At the end, final extension at 72°C for 5 mins was necessary for complete amplification. PCR products were separated and visualized by electrophoresis on a 1.5% ethidium bromide-treated agarose gel (Sigma, UK) according to the standard protocol described by Sambrook et al. [21].

#### 2.5.2. Single-Strand Conformation Polymorphism (SSCP) Analysis and Sequencing

PCR products were denatured using TE buffer ([22]; diluted 1 : 10, pH 8.0). Then, five microliters of diluted solution was mixed with 5 *μ*L of denaturing-loading dye (95% formamide, 4 M urea, 0.1% bromophenol blue, 0.1% Xylene cyanol FF, and 0.5 *μ*L 15% Ficoll) and the mixture was heated to 94°C for 5 mins; then, the mixture was chilled directly on ice for 10 mins [23]. The denaturated PCR samples were subjected to 9% polyacrylamide gel electrophoresis (acrylamide/bisacrylamide = 49 : 1, v/v). At the end, the gel was stained in 100 mL 1 × TBE and 10 *μ*L ethidium bromide (10 mg/mL) and shook for 10 mins to visualize the DNA bands. The gel was placed on a UV trans-illuminator (Stratagene, USA) and pictures were taken with a Polaroid camera (Polaroid MP4 Land Camera).

Bands that abnormally shifted in the SSCP gel compared with their corresponding normal control were considered to harbor somatic mutations. The PCR products that showed mutation using SSCP were sequenced for detection of point mutation.

Amplification products were purified using the QIAquick PCR purification kit (Qiagen, GmbH, Germany). Cycle sequencing of both strands was performed using the BigDye Terminator Kit version 3.1 (Applied Biosystems, Foster City, CA) on an ABI Prism 3730 Genetic Analyzer automated sequencer. Primers for sequencing are described by Morimura et al. [24]. Sequence data was analyzed using the Sequencher 4.1 software package (Gene Codes, MI). If the DNA sequence at a particular location in the DNA differed from the corresponding normal DNA, then it was defined as a somatic mutation.

#### 2.5.3. Comet Assay

The alkaline comet assay was performed as described in detail by Singh et al. [25]. Frosted microscopic slides were dipped into hot 1.0% normal melting point agarose and then the excess agarose was wiped from the underside of the slide. 10 *μ*L of homogenized brain tissue in cold Hank's Balanced Salt Solutions was mixed with 65 *μ*L of 0.5% low melting point agarose at 37°C and covered using a slide cover to spread the samples. The slides were left in lysis solution (2.5 M NaCl, 100 mM Na_2_EDTA, 10 mMTris, NaOH to pH 10.0, 1% Triton-100, and 10% DMSO) for 2 hours at 4°C. The slides were dipped in a coupling jar containing electrophoresis buffer (NaOH, TE buffer) for 20 mins and then electrophoresed at a constant current of 300 mA, for 35 mins. After electrophoresis, the slides were neutralized using Tris-HCl buffer through three washes (5 mins/each wash) at pH 7.5, followed by cold ethyl alcohol for 10 mins, and then left to dry overnight. The slides were stained by using 80 *μ*L ethidium bromide (20 *μ*g/mL) for 20 mins. Then, slides were covered and viewed under an epifluorescence microscope (Zeiss epifluoresent) with an attached CCD camera. Images were saved as electronic files and, for each sample, 50 isolated comets were randomly selected and measured for comet tail length, %DNA in tail, and tail moment using COMETSCORE software based on the definition by Olive and Banánth [26].

#### 2.5.4. Statistical Data Analysis

Data were expressed as the mean ± standard error (M ± SE). Statistical significances of differences between two groups were determined using Student's* t*-test. The post hoc Tukey HSD test was performed to determine which pairs of different sampling time groups are significantly different from each other. The difference between means at the level of *p* < 0.05 was considered as significance. Statistics were carried out using statistical analysis systems (SAS) program.

## 3. Results

Results of TiO_2_ nanoparticles characterization published in previous studies [17] confirmed the rutile-anatase commercial form of nano-TiO_2_ using XRD analysis and evidenced the nanosize of nano-TiO_2_ suspension in water using TEM (46.23 ± 3.45 nm). Moreover, TEM confirmed the polyhedral morphology of the crystallite structure with increasing surface area and activity.

**Figure 1:**
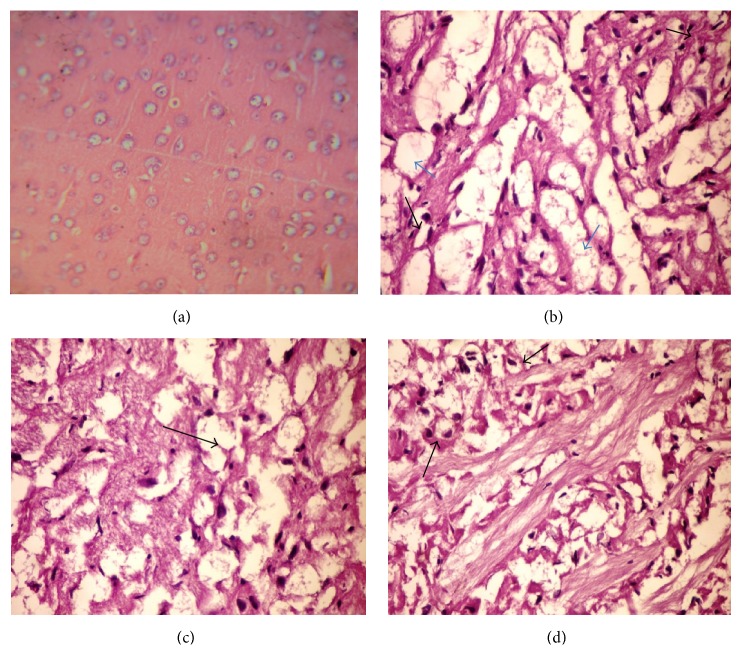
Photomicrograph of brain sections of different groups, in which (a) represents negative control; (b) 24 h group showing extracellular brain edema (blue arrows) and intracellular brain edema (black arrow); (c) 7-day group showing an extracellular edema (arrows); (d), 14-day group showing vacuolation (arrows), respectively. Hematoxylin and eosin staining at 400x.

Figure 1 shows brain sections of different groups, in which (a) represents negative control group; it shows apparently healthy brain cells. TiO_2_NPs (500 mg/kg) treated group sacrificed after 24 h shows spongiosis as an extracellular brain edema (blue arrows), together with intracellular brain edema (black arrow) as shown in Figure 1(b). Moreover, TiO_2_NPs (500 mg/kg) treated group at 7-day sampling time shows an extracellular edema (arrows) (Figure 1(c)), while TiO_2_NPs (500 mg/kg) treated group at 14-day sampling time shows the most damaged brain tissue, represented by vacuolation of nerve cells with peripheral nucleolus and formation of signet ring appearance (Figure 1(d), arrows).

**Figure 2:**
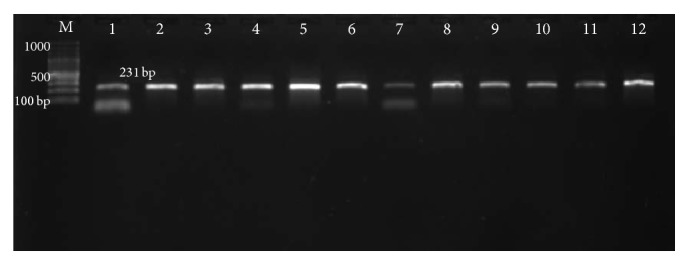
A 1.5% agarose gel separating PCR product for PSEN1 exon 5 (231 bp). Each lane represents an individual animal, in which lane M is low molecular weight DNA marker (100–1500 bp); lanes 1–3, PCR product for negative control group; lanes 4–6; lanes 7–9 and lanes 10–12, PCR products for 24 h; 7-day and 14-day groups, respectively.

Figure 2 represents successful PCR product for negative control and different treated groups at expected molecular weight 231 bp. Primers were specified for PSEN1 gene exon 5 of mouse.

**Figure 3:**
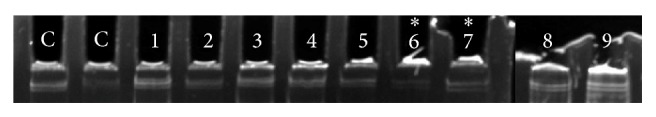
A 9% polyacrylamide gel showing PCR-SSCP for PSEN1 exon 5. Each lane represents an individual animal, in which lane C represents negative control group; lanes 1–3; lanes 4–6 and lanes 7–9 represent PCR-SSCP pattern for 24 h; 7-day and 14-day groups, respectively. (*∗*) symbol referred to mutated samples that are found at lanes 6 and 7 in comparison with the negative control group.

**Figure 4:**
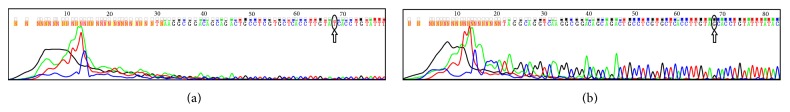
DNA sequence chromatogram of (a) negative control, (b) representative TiO_2_NPs 7-day and 14-day mutated groups, using PSEN1 exon 5 reverse primer, in which point of mutation is circled and indicated by arrow.

Figure 3 represents PCR-SSCP for negative control and TiO_2_NPs treated groups at different sampling time, in which, from each TiO_2_NPs (500 mg/kg) treated group at 7-day and 14-day sampling time, there is one mouse (from three/group) that shows a somatic mutation represented by a band shift in comparison with the negative control group. In another word, one-third of each group has point mutation. While, there is not any difference in the PCR-SSCP pattern of the other treated groups relative to the negative control group. Mutated PCR samples were subjected to direct sequencing to detect point mutation. Direct sequencing shows a point mutation at site 33042 bp, at this site T base was substituted with G base at the same site in all mutated samples in comparison with negative control group (Figure 4). Therefore, this site has a common point mutation in TiO_2_NPs (500 mg/kg) treated groups at sampling time of 7 days or 14 days.

**Figure 5:**
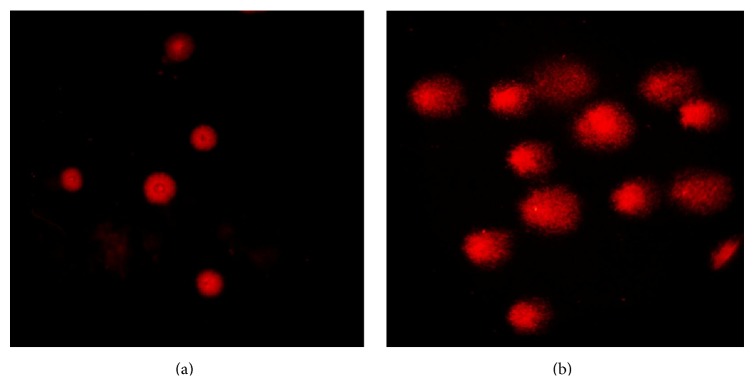
Representative photomicrograph showing (a) typical nuclei of undamaged cells of negative control group and (b) various degrees of DNA damage observed as comets that were seen in all different treated groups.

**Figure 6:**
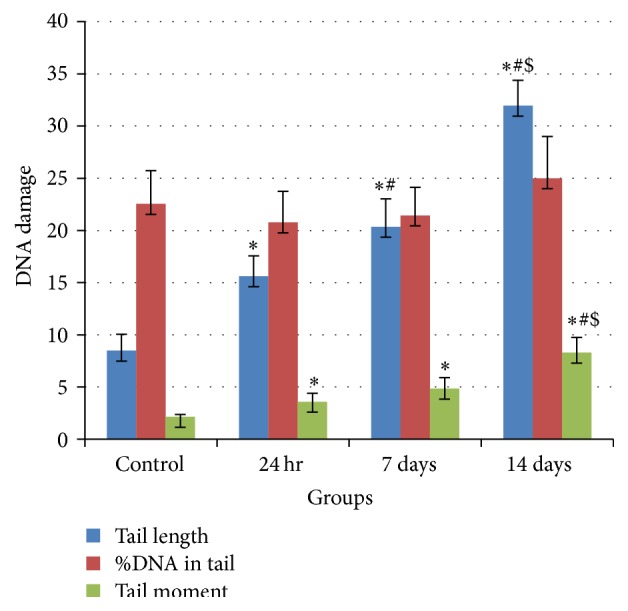
Effect of TiO_2_NPs on the DNA (DNA damage was represented by Comet assay) in mice brain cells. Significant difference (*p* < 0.05) using Student's* t*-test, in which ^*∗*^Statistically compared with negative control group; ^#^Statistically compared with 24 h group; ^$^Statistically compared with 7-day group.

The genotoxic effect of TiO_2_NPs at different sampling time was evaluated by using Comet assay.  Figure 5(a) shows typical nuclei of undamaged cells for negative control group, while Figure 5(b) is a representative photomicrograph for various degrees of DNA damage observed as comets that were seen in all different treated groups. Fifty isolated comets were randomly selected for all groups and measured for comet tail length, %DNA in tail, and tail moment using COMETSCORE software. The selected data (mean ± SE) for all treated groups were compared using Student's* t*-test (significant difference *p* ≤ 0.05). The results show a significant increase in tail length and tail moment for all treated groups in comparison with the negative control group. Except for %DNA in tail, it shows a nonsignificant increase only in 14 days' group, while the other groups show a nonsignificant decrease as shown in Figure 6.

Moreover, Figure 6 shows a significant increase in tail length and tail moment when treated groups were statistically compared with 24 h group, except for tail moment of 7 days' group, while there is a nonsignificant increase in %DNA in tail of treated groups in comparison with 24 h group. In addition, Figure 6 statistically compared 14 days' group with 7 days' group; the results show an increase in tail length, %DNA in tail, and tail moment of 14 days' group; this increase is significant for tail length and tail moment.  Table 1 shows that there is a significant difference between groups 24 hr versus 14 days and 7 days versus 14 days at tail length and tail moment.

## 4. Discussion

The present study reports the genotoxic and mutagenic effect of TiO_2_NPs on brain cells at different sampling time. Moreover, exposure to TiO_2_NPs produces mild to moderate change in the cytoarchitecture of brain tissue. We hypothesized that those different toxic and mutagenic effects might lead to AD incidence.

The present study referred to the most important organ, the brain that might be affected by TiO_2_NPs administration. There are few studies in this point of interest [27]. TiO_2_NPs (500 mg/kg) treatment at different sampling time leads to brain tissue damage that is highly affected at 14 days' sampling time. Those results were in agreement with Block et al. [28], who reported the* in vitro* neurotoxicity of TiO_2_NPs (5 ppm), in which it stimulates BV2 microglia to produce ROS that in turn damages neurons in cultures of brain striatum. In addition, Ma et al. [29] reported the neurotoxic effect of intra-abdominal injections of 5–150 mg/kg nano-TiO_2_ (5 nm) daily for 14 days in female mice, represented by filamentous-shaped neurons and inflammatory cells. Moreover, Li et al. [13] recorded the neurotoxic intratracheal effect of 3.3 mg/kg nano-TiO_2_ (3 nm) once a wk for 4 wks in male mice, represented by exudates, inflammatory infiltration, and necrosis of brain tissue.

The SSCP-pattern followed by direct sequencing revealed the mutagenic effect of TiO_2_NPs at 7 and 14 days' sampling time on PSEN1 gene at exon 5. To the best of our knowledge, this is the first time to detect this point mutation as a result of TiO_2_NPs treatment on this important AD related gene. Previous study reports the gastric mutagenic effect of TiO_2_NPs (at different doses 5, 50, and 500 mg/kg b.w) treatment, in which it induced high mutation frequencies in p53 exons (5–8) in a dose and time dependent manner [17]. PSEN gene is assumed to be the catalytic subunit of *γ*-secretase, and mutations in the PSEN1 and PSEN2 genes are the most common cause of familial AD [30]. Crews and Masliah [31] referred to previous studies that, in familial forms of AD, PSEN mutations result in an increase of amyloid-*β* (A*β*) protein production or aggregation that in turn results in plaque formation and synaptotoxicity. This might confirm our hypothesis that TiO_2_NPs exposure might lead to familial form of AD but further studies will be needed in this point to know if this point mutation will be effective or not.

From the present Comet assay, results report the genotoxic potential of TiO_2_NPs on brain cells. Those results were in agreement with Landsiedel et al. [8]; they published a review that describes various knowledge about genotoxicity investigations on nanomaterials including TiO_2_NPs. They declare the evidence of TiO_2_NPs genotoxicity represented by micronuclei development, as an indicative of chromosomal damage and DNA damage. Comet assay and the detection of* in vitro* mammalian chromosomal aberrations are the most commonly used test systems to assess genotoxicity.

Although TiO_2_NPs have the efficiency to stimulate microglia [28], this may lead to oxidative burst that can be represented by the immediate production and release of superoxide anions (O_2_^−∙^) that convert to multiple ROS such as hydrogen peroxide (H_2_O_2_), hydroxyl radicals, and peroxynitrites. The excess anions can diffuse from the microglial plasma membrane and damage the proteins, lipids, and DNA of neighboring cells, especially neurons, and might lead to neurodegeneration. Therefore, TiO_2_NPs exposure might lead to the incidence of the most common neurodegenerative disorder, Alzheimer's Disease.

## Figures and Tables

**Table 1:** Post hoc Tukey HSD test showing the difference between different group pairs.

Groups	Tail length mean ± SD	Post hoc Tukey HSD *p* value	% DNA in tail mean ± SD	Post hoc Tukey HSD *p* value	Tail moment mean ± SD	Post hoc Tukey HSD *p* value
24 hr	15.62 ± 1.94	0.109	20.76 ± 2.99	0.900	3.60 ± 0.80	0.422
7 days	20.34 ± 2.69		21.44 ± 2.69		4.85 ± 1.05	

24 hr	15.62 ± 1.94	0.001^*∗*^	20.76 ± 2.99	0.319	3.60 ± 0.80	0.005^*∗*^
14 days	31.97 ± 2.40		25.01 ± 4.01		8.30 ± 1.45	

7 days	20.34 ± 2.69	0.002^*∗*^	21.44 ± 2.69	0.431	4.85 ± 1.05	0.023^*∗*^
14 days	31.97 ± 2.40		25.01 ± 4.01		8.30 ± 1.45	

^*∗*^Significant difference (*p* ≤ 0.05) using post hoc Tukey HSD test.

## Abbreviations

TiO_2_NPs: Titanium dioxide nanoparticles
AD: Alzheimer's disease
OS: Oxidative stress
PSEN1: Presenilin 1.
